# Structured and unstructured intraspecific propagule trait variation across environmental gradients in a widespread mangrove

**DOI:** 10.1002/ece3.10835

**Published:** 2024-01-09

**Authors:** Jingjun Yang, Anchi Wu, Jinhua Li, Haihang Wei, Jie Qin, Hongdeng Tian, Donghan Fan, Weidai Wu, Shan Chen, Xin Tong, Xiu Liu

**Affiliations:** ^1^ Guangxi Forestry Research Institute Nanning China; ^2^ Key Laboratory of Vegetation Restoration and Management of Degraded Ecosystem, South China Botanical Garden, Chinese Academy of Sciences Guangzhou China; ^3^ College of Resources and Environment University of Chinese Academy of Sciences Beijing China; ^4^ Qinzhou Forestry Research Institute Qinzhou China; ^5^ School of Ecological and Environmental Sciences East China Normal University Shanghai China; ^6^ Eastern China Conservation Centre for Wild Endangered Plant Resources, Shanghai Chenshan Botanical Garden Shanghai China

**Keywords:** intraspecific trait variation, *Kandelia obovate*, mangrove, propagule shape, propagule size

## Abstract

Increasing studies have shown the importance of intraspecific trait variation (ITV) on ecological processes. However, the patterns and sources of ITV are still unclear, especially in the propagules of coastal vegetation. Here, we measured six hypocotyl traits for 66 genealogies of *Kandelia obovata* from 26 sites and analyzed how ITV in these traits was distributed across geography and genealogy through variance partitioning. We further constructed mixed models and structural equation models to disentangle the effects of climatic, oceanic, and maternal factors on ITV. Results showed that size‐related traits decreased along increasing latitudinal gradients, which was mainly driven by positive regulation of temperature on these traits. By contrast, ITV of shape trait was unstructured along latitudinal gradients and did not show any dependence among environmental variables. These findings indicate that propagule size mainly varied between populations, whereas propagule shape mainly varied between individuals. Our study may provide useful insights into the ITV in propagule from different functional dimensions and on a broad scale, which may facilitate mangrove protection in light of ITV.

## Graphical abstract

Size‐related traits decreased along increasing latitudinal gradients, which was mainly driven by positive regulation of temperature on these traits. By contrast, intraspecific variation in shape trait was unstructured along latitudinal gradients and did not show any dependence of environmental variables. These findings indicate that propagule size mainly varied between populations, whereas propagule shape mainly varied between individuals.

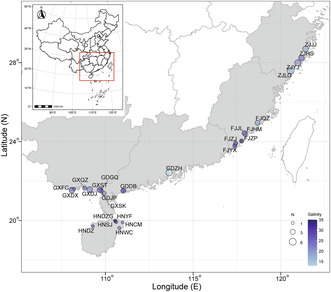

## 1 INTRODUCTION

Despite being recognized as a foundation for the theory of evolution by natural selection, the importance of intraspecific trait variation (ITV) has been neglected over time in ecology (Bolnick et al., 2011). Recently, there has been a resurgence of ecological interest in ITV, stimulated by the proliferating studies that underline the tremendous effects of ITV on community assembly and ecosystem functioning (Cardou et al., 2022; Violle et al., 2012). However, the patterns and sources of ITV in themselves are still unclear (Cope et al., 2022). For example, ITV can be structured at various spatial scales in relation to different drivers. Between‐population ITV tends to be shaped by large‐scale environmental gradients, whereas within‐population ITV is more likely the consequence of heritable differences and/or plastic responses to the local environment (Albert et al., 2010; Martin et al., 2017). Alternatively, ITV can be unstructured if the trait is mainly determined by stochastic processes or constrained by fitness trade‐offs (Kendall & Fox, 2003; Moran et al., 2016). Elucidating ITV distribution across multiple scales is yet crucial for understanding and predicting ecological responses to global changes (Cochrane et al., 2015; Moran et al., 2016).

Trait‐based plant studies to date have largely focused on vegetative traits (e.g., leaf and root functional traits), with less attention placed on seed traits despite the significance of seed traits for plant regeneration and long‐term persistence (Saatkamp et al., 2019). Knowledge of seed traits is particularly urgent for mangroves, which once covered over 200,000 km^2^ of sheltered tropical and subtropical coastlines but nowadays has been disappearing worldwide for decades at an extremely high rate (Duke et al., 2007; Friess et al., 2019; Giri et al., 2015). Due to the property of low species richness and redundancy, mangrove degradation is always followed by pronounced losses of ecological multifunctionality (Arifanti et al., 2022; Donato et al., 2011; Feller et al., 2010). Such situation emphasizes mangrove recruitment and thereby calls for characterizing seed/propagule traits that represent key dimensions of the “regeneration niche” (sensu Grubb, 1977) in mangroves (Feller et al., 2010; Peterson & Bell, 2012).

Many mangroves (e.g., Rhizophoraceae and Avicenniaceae) have evolved a special reproductive strategy of viviparous seeds (germinating precociously while attached to the maternal plant), probably reflecting an adaptation to the salty and flooding intertidal environments (Feller et al., 2010). Due to the lack of seed dormancy, mangrove forests destroyed by severe disturbances such as hurricanes and tsunamis may not have local seed reserves, necessitating recolonization through long‐distance seed dispersal from relatively undisturbed populations (Nettel & Dodd, 2007). This is particularly the case for Rhizophoraceae forests, as Rhizophoraceae species lack the capacity of resprouting from damaged trees as Avicenniaceae species (Baldwin et al., 2001). Therefore, dispersal/retention is a key dimension of propagule functions where focal traits should be targeted for mangroves (Van der Stocken et al., 2019). Whether dispersed or retained, establishment is a prerequisite for any propagule to contribute to the regeneration, representing another critical dimension of seed trait (Krauss et al., 2008).

Most ITV studies in mangroves, though few in absolute number, have investigated propagule traits (e.g., size and weight) in relation to establishment, rarely considering the dispersal/retention dimension (Saenger & West, 2018; Yang et al., 2020; Zhu et al., 2021). These studies showed that temperature and/or precipitation may have given rise to structured ITV in mangroves across geographic gradients (Saenger & West, 2018; Yang et al., 2020; Zhu et al., 2021), a pattern commonly reported in terrestrial forests (Kumordzi et al., 2019). Nevertheless, as mangroves are coastal vegetation, their trait variation may also be shaped by oceanic factors including salinity and tidal currents (Richards et al., 2021; Sousa et al., 2007). Additionally, maternal plants can also affect propagule traits, through both genetically fixed differences and environmentally induced transgenerational plasticity (Alam et al., 2018; Cochrane et al., 2015). However, the relative importance of these abiotic and biotic factors in shaping the distribution of ITV across multiple scales, and whether the patterns differ between the propagule function dimensions (establishment vs. dispersal/retention) are still poorly understood.

Here we use *Kandelia obovata* as the model species to investigate the issues. *Kandelia obovata* (Rhizophoraceae) is the most cold‐tolerant true‐mangrove species and has a wide latitudinal distribution along the southeast coast of China (Sheue et al., 2003; Wang et al., 2011), providing an ideal system for studying the ITV in mangroves. Using a stratified sampling design across a 9° latitudinal gradient, we analyzed the structure of intraspecific variability for five hypocotyl functional traits in relation to propagule dispersal/retention and establishment. The stratified sampling design and contrasting environmental conditions allowed us to address the following questions: (i) How is ITV structured spatially (between populations, maternal trees, hypocotyls)? (ii) What is the major driver (climatic, oceanic, or maternal factors) shaping the distribution of ITV? Based on the results from previous studies (Saenger & West, 2018; Zhu et al., 2021), the variability of traits on the establishment axis is expected to be higher between populations than within populations and predominantly shaped by abiotic factors. By contrast, the trait on the dispersal/retention axis, likely more reflecting a trade‐off between post‐disturbance recolonization and local recruitment (Sousa et al., 2007; Van der Stocken et al., 2019), may be less structured or even unstructured with regard to particular environmental gradients.

## 2 MATERIALS AND METHODS

### 2.1 Species, study sites, and sample collection

**FIGURE 1:**
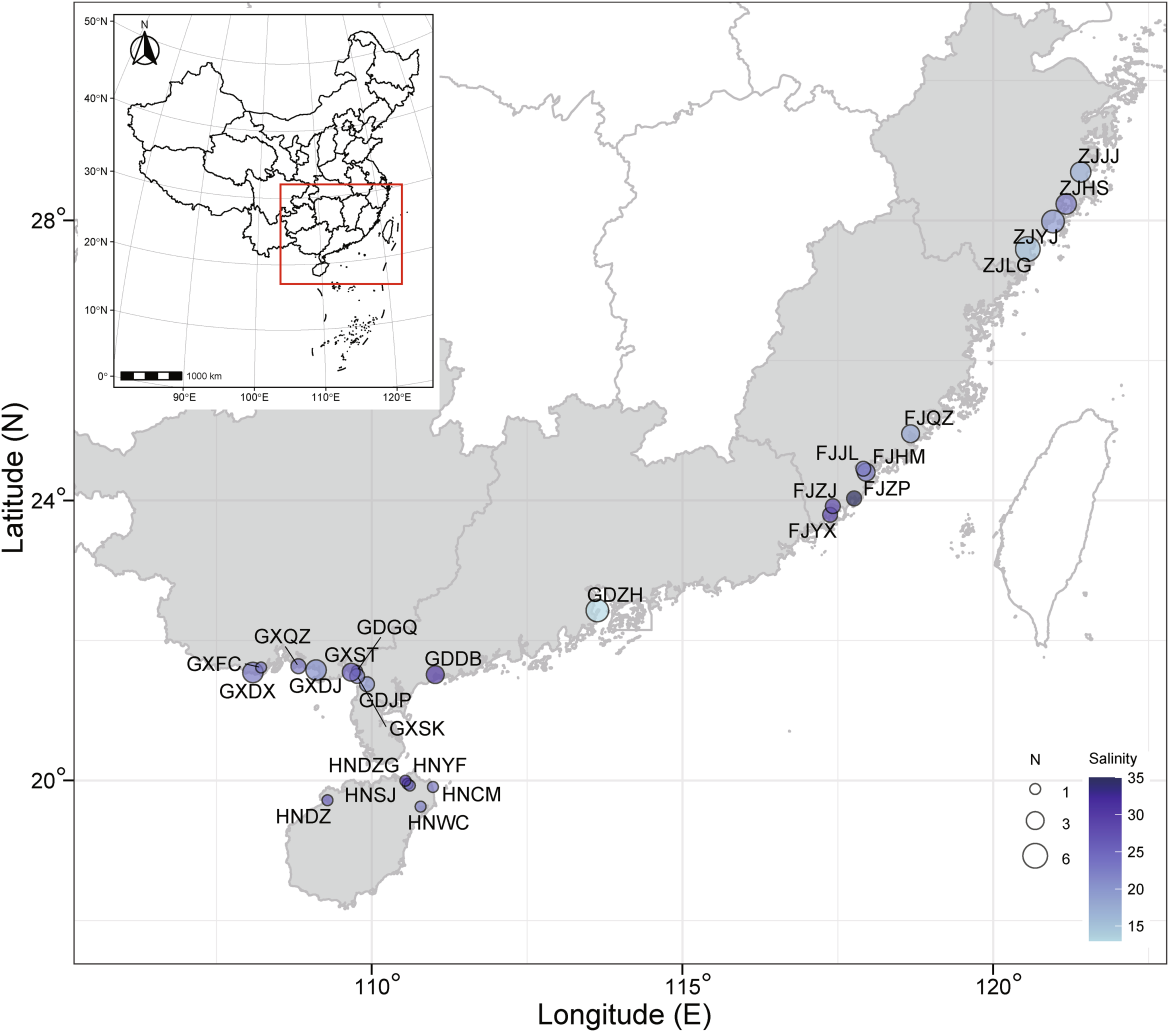
Sampling sites of *Kandelia obovata* hypocotyl on the south coast of China. The color gradient indicates the salinity (‰) of surface seawater. Circle size is proportional to sampling size of maternal trees (*N*).

*Kandelia obovata* was first reported by Sheue et al. (2003), which was mainly different from the relative species *K. candel* (L.) Druce in leaf shape and cold tolerance. The two species are well‐differentiated sets of geographical populations separated by the South China Sea. In China, *K. obovata* ranges from Hainan, Guangxi, Guangdong, Fujian, Zhejiang and Taiwan. Mature hypocotyls of *K. obovata* were collected during 2020–2021 from 26 sites along the coastline of southern China, spanning from 19°37′ N to 28°41′ N in latitude and from 108°05′ E to 121°24′ E in longitude (Figure 1). This geographic range provides wide climatic gradients in annual average precipitation (1102–2373 mm, mean 1578 mm), annual mean temperature (14.7–24.7°C, mean 21.1°C), and surface seawater salinity (13‰–35‰, mean 21.8‰). We sampled 1–6 maternal trees from each site (66 trees in total), depending on the population size. To ensure genetic independence, the sampled trees were spaced at least 30 m apart according to Geng et al. (2008). Each maternal tree was thus considered as a single genealogy. We randomly collected 30 hypocotyls from each tree for the study.

### 2.2 Trait measurement for hypocotyls and maternal trees

We measured traits including fresh weight (FW), fresh length (FL), maximum transverse diameter (TD_max_), and minimum transverse diameter (TD_min_) for each hypocotyl. We also calculated the shape index of hypocotyl as the ratio of maximum transverse diameter to the minimum (RTD). This index is important for propagule floating because the slender morphology propagule disperses fast (De Ryck et al., 2012). We measured height (H) and diameter at breast height (DBH) for maternal trees, and transformed them into aboveground biomass (AGB) using an allometric model of tropical wood tree: AGB = 0.0673 × (*ρ* × DBH^2^ × H)^0.976^ (Chave et al., 2014), where wood density *ρ* is set as 0.57 g/cm^3^ (Jiang, 2014). The AGB is a comprehensive outcome of the interaction between the genotype and the environment experienced by maternal trees, thereby capturing multiple facets of how maternal trees can affect the progeny. We thus used AGB as a synthesis indicator to predict potential effects of maternal performance on hypocotyl traits.

### 2.3 Access to climate and tide data

We extracted the data of each site from the WorldClim dataset (Hijmans et al., 2005) for mean annual temperature (MAT) and mean annual precipitation (MAP), and from Global Tidal Forecasting Service (http://global‐tide.nmdis.org.cn/) for tide and tidal current datasets. Specifically, the data on the highest tide, the lowest tide, the fastest tidal current, and the slowest tidal current were obtained from the nearest tide station. Since the annual average of the tide data is not available, we used the data of 4 months (January, April, July, and October) to represent the whole year. We calculated the annual highest tide, annual lowest tide, annual fastest tide current, and annual slowest tide current. We also measured sea surface salinity at each site using the Portable Refractometer (Apu, China).

### 2.4 Data analysis

Bivariate analysis with ordinary least squares linear regression and quadratic regression were used to quantify how hypocotyl trait values varied with latitudinal gradients and biotic/abiotic factors. Because of high correlations among most hypocotyl traits (*r* > .36, *p* < .001), we performed a principal component analysis with multiple traits using the “princomp” function in R 4.1.3 (R Core Team, 2022) and used the two first principal component axes to represent the hypocotyl traits.

To evaluate how environmental factors, maternal plants, or inherent factors explained variation in hypocotyl traits, we used a nested analysis of variance coupled with variance partitioning techniques (Martin et al., 2017). We carried out linear mixed model for the first axis (PC1) and RTD by using the “lme” function in “nlme” R package (Pinheiro et al., 2022). In each model, all nested levels (i.e., site > genealogy > within [individual]) were entered as sequential random effects and the intercept was the only estimated fixed effect. We then used the “varcomp” function in the “ape” R package (Paradis et al., 2004) to calculate the variance components associated with each nested level.

To quantify how hypocotyl traits were affected by climatic factors, oceanic factors, and maternal performance, we implemented linear mixed models using the “lme” function in the R package “nlme.” The fixed‐effect terms included the climatic, oceanic, and maternal variables. To account for additional variation potentially caused by some missing site‐specific effects (e.g., other environmental factors), and that caused by other maternal effects uncaptured by AGB, we treated sampling site and tree genealogy as random factors. All variables were standardized before the modeling such that each variable had a mean of zero and a standard deviation of one. To reduce the adverse influence of multicollinearity, we removed multicollinear variables until the variance inflation factors of all variables in the model were less than three (Ouyang et al., 2019). Both primary and quadratic mixed models were considered, and only the better‐fitted model was shown (based on the Akaike information criteria). We calculated the variance inflation factor using the R package “car” (Fox & Monette, 2019). The pseudo‐*R* ^2^ was calculated using the function “r.squaredGLMM” in the R package “MuMIn” (Bartoń, 2022), to represent the variance explained by the fixed effect in the linear mixed model. The effect sizes of fixed factors were measured by the regression coefficients in the linear mixed model.

Structural equation modeling was used to disentangle direct and indirect effects of all predictive factors on hypocotyl traits. After standardizing all variables, multicollinear variables were removed based on variance inflation factor. We first considered a full model that included all variables and all reasonable pathways. Non‐significant pathways were then sequentially removed, unless the pathways were biologically informative. The removing and adding of pathways were repeated until both *p*_*χ*2‐test_ ≥ .05 (i.e., no significant difference between model predictions and the observed data) and root mean square error of approximation <.08 were reached (Wu et al., 2022). The structural equation modeling was performed using the “lavaan” R package (Rosseel, 2012).

## 3 RESULTS

### 3.1 Patterns of ITV in hypocotyl along latitudinal gradients

**FIGURE 2:**
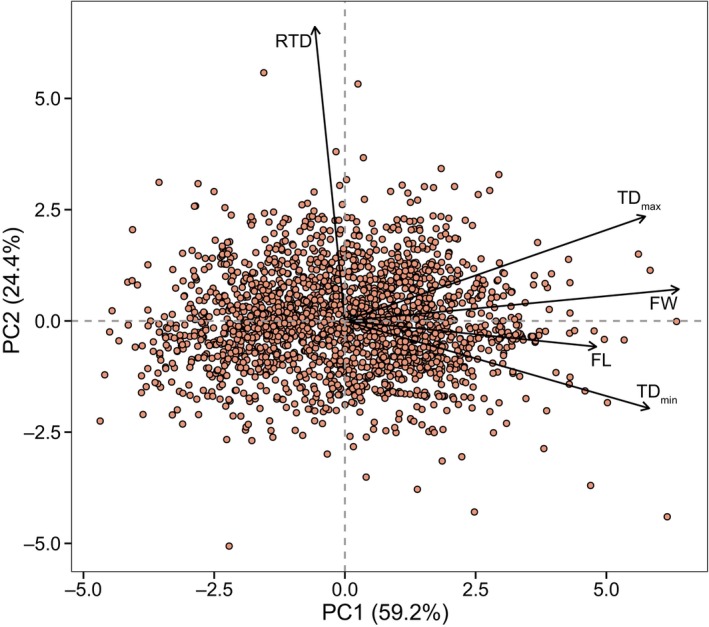
Principal component analysis of hypocotyl traits. FW, fresh weight; FL, fresh length; TD_max_, maximum transverse diameter; TD_min_, minimum transverse diameter; RTD, ratio of TD_max_ to TD_min_.

**FIGURE 3:**
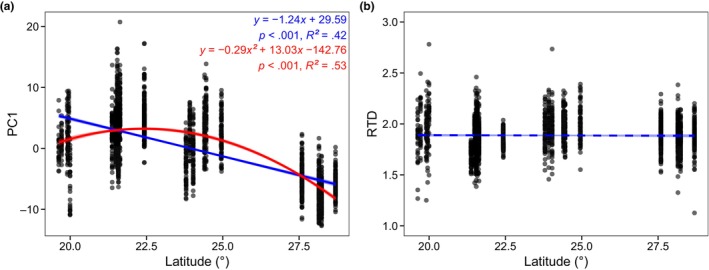
Patterns of hypocotyl intraspecific traits variations along latitudinal gradients. (a) Relationship between PC1 and latitude. (b) Relationship between RTD and latitude. Blue line indicates ordinary least squares linear regression and red line indicates quadratic regression. Solid line indicates statistical significance (*p* < .05) and dashed line indicates statistical non‐significance (*p* > .05). Filled area means standard error. PC1, the first principal component. RTD, ratio of TD_max_ to TD_min_.

The first two principal component axes (associated eigenvalues >1) accounted for 83.6% of the total variation among all these traits, where the PC1 mainly reflected hypocotyl mass and size (including fresh weight, fresh length, maximum transverse diameter, and minimum transverse diameter), and the second axis was primarily determined by the shape index (RTD; Figure 2). The PC1 decreased broadly with increasing latitudinal gradients (*R* ^2^ = .42, *p* < .001), yet statistically quadratic regression had a better explanation for their relationship (*R* ^2^ = .53, *p* < .001; Figure 3a). Intriguingly, RTD did not show any significant correlation with latitude (*p* = .6; Figure 3b).

### 3.2 Sources of ITV in hypocotyl

**FIGURE 4:**
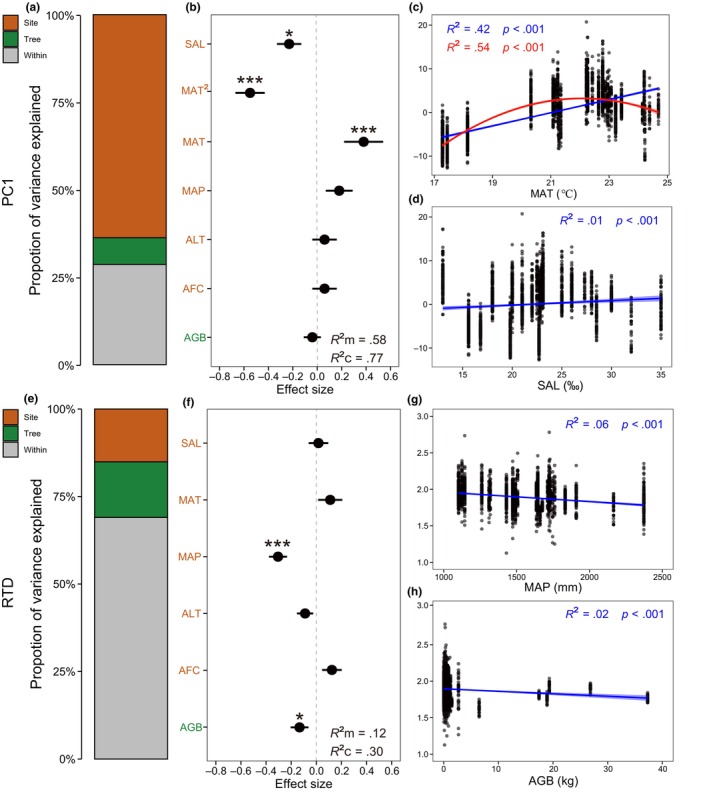
Sources of hypocotyl intraspecific traits variations. (a, e) Variance partitioning of PC1 and RTD across three nested levels of organization. (b, f) Summary of the linear mixed‐effect modeling for multiple biotic and abiotic factors on PC1 and RTD. Data are presented as coefficients ± standard errors of the estimated effect sizes. *R* ^2^m represents marginal *R* ^2^ and *R* ^2^c represents conditional *R* ^2^. *p < .05; ***p < .001. (c, d, g, h) The relationships between significant biotic and abiotic factors and PC1 and RTD. Blue line indicates ordinary least squares linear regression and red line indicates quadratic regression. Filled area means standard error. SAL, sea surface salinity; MAT, mean annual temperature; MAP, mean annual precipitation; AFC, annual fastest tide current; ALT, annual lowest tide; AGB, aboveground biomass. PC1, the first principal component. RTD, ratio of TD_max_ to TD_min_.

Variance partitioning showed that the majority of variation in PC1 (63.75%) existed at the site level, with much less variation explained by genealogy (7.62%) or by individual (28.63%; Figure 4a). In contrast, the variability of hypocotyl shape did not appear to be structured either spatially or genealogically, as shown by the small part of RTD variation attributed to site (15.10%) or genealogy (15.87%). Variability within individuals represented a large part of the variance in RTD (69.03%; Figure 4e).

The linear mixed model indicated that 58% of the variation in PC1 could be explained by the predictive variables (quadratic mixed model). Specifically, the quadratic term of MAT (i.e., MAT^2^) had the strongest negative effect on PC1 (standardized coefficient =  −0.55 ± 0.12, *p* < .001), while MAT had a significant positive effect on PC1 (standardized coefficient = 0.38 ± 0.16, *p* < .001). Sea surface salinity had a significant negative effect on PC1 (−0.21 ± 0.09, *p* < .05; Figure 4b). The bivariate relationships between MAT and PC1 (ordinary least squares linear regression: *R* ^2^ = .42; quadratic regression: *R* ^2^ = .54) were much stronger than those between salinity and PC1 (*R* ^2^ = .01; Figure 4c,d). In contrast, all these predictive factors together explained only 12% of the variation in the shape index RTD, though significant effects of MAP (standardized coefficient = −0.30 ± 0.07) and AGB (standardized coefficient = −0.15 ± 0.07) were indicated by the model (Figure 4f). Bivariate analyses also showed a significant correlation between MAP or AGB and RTD, yet with very low explanation of the model (*R* ^2^ = .06 and *R* ^2^ = .02, respectively; Figure 4g,h).

### 3.3 Direct and indirect effects on hypocotyl traits

**FIGURE 5:**
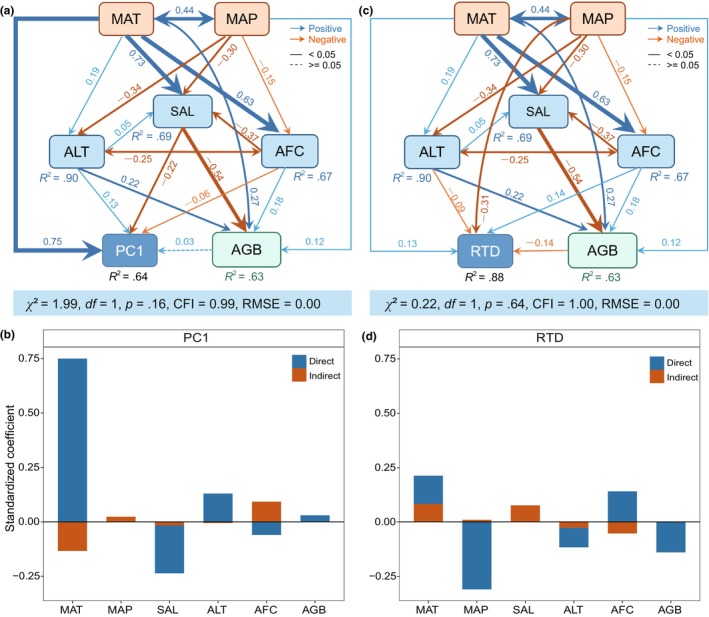
Direct and indirect effects of biotic and abiotic factors on hypocotyl intraspecific trait variations. (a, c) Structural equation models showing the relationships among biotic, abiotic factors and PC1 and RTD. Blue and red arrows indicate positive and negative relationships, respectively. Solid and dashed lines indicate significant (*p* < .05) and non‐significant relationships, respectively. Numbers near the pathway arrow indicate the standard path coefficients. *R* ^2^ represents the proportion of variance explained. (b, d) Standardized effects derived from structural equation models of PC1 and RTD. Blue and red bars indicate direct and indirect effect, respectively The relevant indicators are abbreviated as: PC1, the first principal component; RTD, ratio of TD_max_ to TD_min_; AGB, aboveground biomass; SAL, sea surface salinity; AFC, annual fastest tide current; ALT, annual lowest tide; MAT, mean annual temperature; MAP, mean annual precipitation; CFI, comparative fit index; RMSE, root mean square error of approximation.

We further used structural equation modeling to disentangle the direct and indirect effects of climatic, oceanic, and maternal factors on hypocotyl traits. Overall, these variables could explain 64% and 88% of the variation in PC1 and RTD, respectively. MAT played the strongest role in directly shaping PC1 (standardized path coefficients, *β* = .75). Sea surface salinity (*β* = −.22) was the main oceanic factor that directly affected PC1. Tide effects of annual lowest tide and annual fastest tide current on PC1 were much weaker than that of MAT. MAP had no direct effect and AGB had a weak effect on PC1 (Figure 5a,b). In contrast, all the explanatory variables, except MAP (*β* = −.31), had a weak direct effect on RTD (|*β*| < .15). Compared with the effects of climatic factors on PC1, effects of climatic factors were much smaller on RTD (Figure 5c,d).

## 4 DISCUSSION

Increasing studies have showed the importance of ITV on ecological processes. However, the patterns and sources of ITV in themselves are still unclear (Cope et al., 2022). There are large ITVs in mangroves due to the complex environment but are less concerned on propagule (Feller et al., 2010; Peterson & Bell, 2012). It is urgent to study the ITV in mangrove propagule at the background of global changes. We analyzed the relationship between biotic and abiotic factors and hypocotyl traits on a large scale for a typical viviparous mangrove species, *Kandelia obovata*. We found that hypocotyl size‐related traits were mainly structured between populations, a pattern mainly shaped by climate. However, hypocotyl shape trait was almost unstructured either spatially or genealogically, with its variance predominantly attributed to the within‐individual level. This pattern may suggest that variation in hypocotyl shape is independent of environmental variables. Our study provides insights into the ITV mechanism of hypocotyl traits in mangrove plants.

### 4.1 Structured trait variation in mangrove hypocotyl

Previous studies have showed the presence of ITV in propagule size, especially for the genera *Rhizophora*, *Bruguiera*, *Kandeli*, and *Ceriops* (Tomlinson, 2016). Our results showed that variation in PC1 was significantly negatively correlated with latitude, which is similar to the results of a previous study (Yang et al., 2020). Nevertheless, an inverse relationship has been reported in a black mangrove (*Avicennia marina*), where small propagules were found in the northern (low latitude) populations of Australia yet larger propagules in the southern populations (Saenger & West, 2018). The opposite patterns of structured ITV are possibly due to the different strategies for improving fitness between true viviparous and cryptic viviparous mangrove species. Moreover, genetic analyses also suggested that differences evident in populations of *A. marina* at the different sites of its range are explained as evolutionary adaptations in response to the local physical, climatic, or biological characteristics (Maguire et al., 2000). Therefore, ITV in PC1 was influenced by the local biotic and abiotic factors at different sites.

We found that climate (especially temperature) was the main driver of intraspecific variation in PC1. In general, temperature and precipitation have a negative relation with latitude (Hijmans et al., 2005), while oceanic factors such as salinity and tides have little to do with latitude. It is straightforward and intuitive that more materials can be accumulated in hypocotyl under higher temperature. Precipitation had little effect on PC1, probably because mangrove plants have adapted to tidal environments and are able to obtain fresh water from seawater (Parida & Jha, 2010).

Although oceanic factors were not found to be the main factors affecting the variation in hypocotyl traits, salinity did have a significant effect on PC1 variation. Salinity has long been considered an important factor limiting the growth and distribution of mangroves (Chen & Ye, 2014; Richards et al., 2021), and negative correlations between the lengths and weights of mangrove propagules and sea salinity have been reported (Alam et al., 2018). In addition, salt stress may also be a factor that shapes the evolution of vivipary in mangrove plants. The hypocotyl of viviparous seeds contains a large amount of nutrients and water, which are necessary for the growth of newly colonized seedlings, thus avoiding the vulnerable life stage (Joshi, 1933; Zhou et al., 2016). When salinity is too high, maternal tree will invest more energy to against salt stress (Alam et al., 2018; Parida et al., 2004), which may reduce the investment on the hypocotyl and eventually lead to variation in its traits. Tide is an important factor in mangrove growth and hypocotyl dispersal process (Clarke et al., 2001; Duke et al., 1998; Van der Stocken et al., 2015; Zhang et al., 2021). However, the effect of tides on the variation in hypocotyl traits was marginal in our study. A possible reason is that previous studies have only considered the influence of tidal effects, rather than the combination of multi‐biotic and abiotic effects.

### 4.2 Unstructured trait variation in mangrove hypocotyl

It is interesting that the shape index RTD did not vary with latitude and was much less affected by abiotic and biotic factors than size‐related traits. Few previous studies have focused on the variation in propagule shape at large scales. This lack may be due to the limited distribution areas of other true viviparous mangroves, such as *Bruguiera sexangular*, *B. gymnorrhiza*, and *Rhizophora stylosa* (Wu et al., 2018). Nevertheless, if global warming continues and these thermophilic mangroves expand to higher latitudes, we may also expect unstructured RTD along latitude gradients for their hypocotyls, because they share very similar reproductive strategies as *K. obovata*. By contrast, cryptic viviparous mangroves (e.g. *Avicennia marina*, *Aegiceras corniculatum*) may not show such a pattern, because their propagules are much smaller and even have no elongated hypocotyl (Zhang et al., 2021).

Many studies have emphasized the importance of maternal effects on the variation in offspring traits (Alam et al., 2018; Cochrane et al., 2015; Galloway, 2005), but the strength of maternal effect on ITV was minor in this study. As we only recorded the height and DBH of maternal plants, which reflects the size and age may have less to do with morphology than with yield (Hangelbroek & Santamaria, 2004). Rather, heredity may be the major contributor to maternal effects. In general, genetic diversity is low in natural mangrove populations (Hodel et al., 2018; Richards et al., 2021), but high epigenetic diversity may maintain the high plasticity of traits in mangrove (Mounger et al., 2021). This may explain why the ITV in RTD was mainly attributed to the within‐individual level. Additionally, when traits are related to plant fitness trade‐offs, trait variation may be independent among environmental variables (Bonte et al., 2012). For example, variation in reproductive traits of conifer tree was mainly explained at the scale of individual trees in the western United States (Vasey et al., 2022). This hypothesis needs to be further studied in coastal vegetation.

Due to the lack of dormant seed periods, true mangrove species do not have seed banks and are difficult to regenerate after anthropogenic disturbances or natural disasters (Nettel & Dodd, 2007). Therefore, the collection of germplasm resources and artificial cultivation of true‐viviparous mangrove species are particularly important, especially under global climate changes. High ITV represents high trait diversity and plant may also have higher adaptive capacity (Moran et al., 2016). But if RTD determines some important ecological function (e.g., dispersal), then the low ITV may not allow the mangroves adapt to global change quickly, which in turn affects the mangrove's natural regeneration and persistence. Thus, we recommend that the ITV in hypocotyl should be considered in germplasm collection and restoration so as to facilitate the regeneration of mangrove populations. Future studies that combine biotic and abiotic factors will also help us to have a comprehensive understanding of the trait variation in mangroves and to better protect mangroves.

## AUTHOR CONTRIBUTIONS

**Jingjun Yang:** Conceptualization (equal); data curation (equal); formal analysis (equal); methodology (equal); writing – original draft (equal); writing – review and editing (equal). **Anchi Wu:** Conceptualization (equal); data curation (equal); formal analysis (equal); methodology (equal); writing – original draft (equal); writing – review and editing (equal). **Jinhua Li:** Investigation (equal); project administration (equal); writing – review and editing (equal). **Haihang Wei:** Investigation (equal); project administration (equal); writing – review and editing (equal). **Jie Qin:** Data curation (equal); investigation (equal); methodology (equal). **Hongdeng Tian:** Data curation (equal); investigation (equal); methodology (equal). **Donghan Fan:** Data curation (equal); investigation (equal); methodology (equal). **Weidai Wu:** Data curation (equal); investigation (equal); methodology (equal). **Shan Chen:** Methodology (equal); software (equal); validation (equal); visualization (equal); writing – review and editing (equal). **Xin Tong:** Conceptualization (equal); formal analysis (equal); supervision (equal); writing – original draft (equal); writing – review and editing (equal). **Xiu Liu:** Conceptualization (equal); funding acquisition (equal); project administration (equal); supervision (equal); writing – original draft (equal); writing – review and editing (equal).

## CONFLICT OF INTEREST STATEMENT

The authors declare that they have no competing interests.

## Data Availability

Raw data that support the findings of this study are openly available in DRYAD at https://doi.org/10.5061/dryad.z8w9ghxjt.

## References

[ece310835-bib-0001] Alam, M. R. , Mahmood, H. , Khushi, M. L. R. , & Rahman, M. M. (2018). Adaptive phenotypic plasticity of *Avicennia officinalis* L. across the salinity gradient in the Sundarbans of Bangladesh. Hydrobiologia, 808(1), 163–174. 10.1007/s10750-017-3420-z

[ece310835-bib-0002] Albert, C. H. , Thuiller, W. , Yoccoz, N. G. , Soudant, A. , Boucher, F. , Saccone, P. , & Lavorel, S. (2010). Intraspecific functional variability: Extent, structure and sources of variation. Journal of Ecology, 98(3), 604–613. 10.1111/j.1365-2745.2010.01651.x

[ece310835-bib-0003] Arifanti, V. B. , Kauffman, J. B. , Ilman, M. , Tosiani, A. , & Novita, N. (2022). Contributions of mangrove conservation and restoration to climate change mitigation in Indonesia. Global Change Biology, 28(15), 4523–4538. 10.1111/10.1111/gcb.16216 35470521 PMC9325550

[ece310835-bib-0004] Baldwin, A. , Egnotovich, M. , Ford, M. , & Platt, W. (2001). Regeneration in fringe mangrove forests damaged by hurricane Andrew. Plant Ecology, 157(2), 151–164. 10.1023/A:1013941304875

[ece310835-bib-0005] Bartoń, K. (2022). MuMIn: Multi‐Model Inference. R package version 1.46.0 [online]. https://CRAN.R‐project.org/package=MuMIn

[ece310835-bib-0006] Bolnick, D. I. , Amarasekare, P. , Araújo, M. S. , Bürger, R. , Levine, J. M. , Novak, M. , Rudolf, V. H. W. , Schreiber, S. J. , Urban, M. C. , & Vasseur, D. A. (2011). Why intraspecific trait variation matters in community ecology. Trends in Ecology and Evolution, 26(4), 183–192. 10.1016/j.tree.2011.01.009 21367482 PMC3088364

[ece310835-bib-1001] Bonte, D. , Van Dyck, H. , Bullock, J. M. , Coulon, A. , Delgado, M. , Gibbs, M. , Lehouck, V. , Matthysen, E. , Mustin, K. , Saastamoinen, M. , Schtickzelle, N. , Stevens, V. M. , Vandewoestijne, S. , Baguette, M. , Barton, K. , Benton, T. G. , Chaput‐Bardy, A. , Clobert, J. , Dytham, C. , … Travis, J. M. J. (2012). Costs of dispersal. Biological Reviews, 87(2), 290–312. 10.1111/j.1469-185x.2011.00201.x 21929715

[ece310835-bib-0007] Cardou, F. , Munson, A. D. , Boisvert‐Marsh, L. , Anand, M. , Arsenault, A. , Bell, F. W. , Bergeron, Y. , Boulangeat, I. , Delagrange, S. , Fenton, N. J. , Gravel, D. , Hamel, B. , Hébert, F. , Johnstone, J. F. , Kumordzi, B. B. , Macdonald, S. E. , Mallik, A. , McIntosh, A. C. S. , McLaren, J. R. , … Aubin, I. (2022). Above‐ and belowground drivers of intraspecific trait variability across subcontinental gradients for five ubiquitous forest plants in North America. Journal of Ecology, 110, 1590–1605. 10.1111/1365-2745.13894

[ece310835-bib-0008] Chave, J. , Rejou‐Mechain, M. , Burquez, A. , Chidumayo, E. , Colgan, M. S. , Delitti, W. B. , Duque, A. , Eid, T. , Fearnside, P. M. , Goodman, R. C. , Henry, M. , Martínez‐Yrízar, A. , Mugasha, W. A. , Muller‐Landau, H. C. , Mencuccini, M. , Nelson, B. W. , Ngomanda, A. , Nogueira, E. M. , Ortiz‐Malavassi, E. , … Vieilledent, G. (2014). Improved allometric models to estimate the aboveground biomass of tropical trees. Global Change Biology, 20(10), 3177–3190. 10.1111/gcb.12629 24817483

[ece310835-bib-0009] Chen, Y. P. , & Ye, Y. (2014). Early responses of *Avicennia marina* (Forsk.) Vierh. To intertidal elevation and light level. Aquatic Botany, 112, 33–40. 10.1016/j.aquabot.2013.07.006

[ece310835-bib-0010] Clarke, P. J. , Kerrigan, R. A. , & Westphal, C. J. (2001). Dispersal potential and early growth in 14 tropical mangroves: Do early life history traits correlate with patterns of adult distribution? Journal of Ecology, 89(4), 648–659. 10.1046/j.0022-0477.2001.00584.x

[ece310835-bib-0011] Cochrane, A. , Yates, C. J. , Hoyle, G. L. , & Nicotra, A. B. (2015). Will among‐population variation in seed traits improve the chance of species persistence under climate change? Global Ecology and Biogeography, 24(1), 12–24. 10.1111/geb.12234

[ece310835-bib-0012] Cope, O. L. , Burkle, L. A. , Croy, J. R. , Mooney, K. A. , Yang, L. H. , & Wetzel, W. C. (2022). The role of timing in intraspecific trait ecology. Trends in Ecology and Evolution, 37(11), 997–1005. 10.1016/j.tree.2022.07.003 35918208

[ece310835-bib-0013] De Ryck, D. J. R. , Robert, E. M. R. , Schmitz, N. , van der Stocken, T. , Di Nitto, D. , Dahdouh‐Guebas, F. , & Koedam, N. (2012). Size does matter, but not only size: Two alternative dispersal strategies for viviparous mangrove propagules. Aquatic Botany, 103, 66–73. 10.1016/j.aquabot.2012.06.005

[ece310835-bib-0014] Donato, D. C. , Kauffman, J. B. , Murdiyarso, D. , Kurnianto, S. , Stidham, M. , & Kanninen, M. (2011). Mangroves among the most carbon‐rich forests in the tropics. Nature Geoscience, 4(5), 293–297. 10.1038/Ngeo1123

[ece310835-bib-0015] Duke, N. C. , Ball, M. C. , & Ellison, J. C. (1998). Factors influencing biodiversity and distributional gradients in mangroves. Global Ecology and Biogeography, 7(1), 27–47. 10.2307/2997695

[ece310835-bib-0016] Duke, N. C. , Meynecke, J. O. , Dittmann, S. , Ellison, A. M. , Anger, K. , Berger, U. , Cannicci, S. , Diele, K. , Ewel, K. C. , Field, C. D. , Koedam, N. , Lee, S. Y. , Marchand, C. , Nordhaus, I. , & Dahdouh‐Guebas, F. (2007). A world without mangroves? Science, 317(5834), 41–42. 10.1126/science.317.5834.41b 17615322

[ece310835-bib-0017] Feller, I. C. , Lovelock, C. E. , Berger, U. , McKee, K. L. , Joye, S. B. , & Ball, M. C. (2010). Biocomplexity in mangrove ecosystems. Annual Review of Marine Science, 2, 395–417. 10.1146/annurev.marine.010908.163809 21141670

[ece310835-bib-0018] Fox, J. , & Monette, G. (2019). An {R} companion to applied regression (3rd ed.). Sage https://socialsciences.mcmaster.ca/jfox/Books/Companion/

[ece310835-bib-0019] Friess, D. A. , Rogers, K. , Lovelock, C. E. , Krauss, K. W. , Hamilton, S. E. , Lee, S. Y. , Lucas, R. , Primavera, J. , Rajkaran, A. , & Shi, S. (2019). The state of the world's mangrove forests: Past, present, and future. Annual Review of Environment and Resources, 44(1), 89–115. 10.1146/annurev-environ-101718-033302

[ece310835-bib-0020] Galloway, L. F. (2005). Maternal effects provide phenotypic adaptation to local environmental conditions. New Phytologist, 166(1), 93–99. 10.1111/j.1469-8137.2004.01314.x 15760354

[ece310835-bib-0021] Geng, Q. F. , Lian, C. L. , Goto, S. , Tao, J. M. , Kimura, M. , Islam, M. D. S. , & Hogetsu, T. (2008). Mating system, pollen and propagule dispersal, and spatial genetic structure in a high‐density population of the mangrove tree *Kandelia candel* . Molecular Ecology, 17(21), 4724–4739. 10.1111/j.1365-294X.2008.03948.x 19140988

[ece310835-bib-0022] Giri, C. , Long, J. , Abbas, S. , Murali, R. M. , Qamer, F. M. , Pengra, B. , & Thau, D. (2015). Distribution and dynamics of mangrove forests of South Asia. Journal of Environmental Management, 148, 101–111. 10.1016/j.jenvman.2014.01.020 24735705

[ece310835-bib-0023] Grubb, P. J. (1977). Maintenance of species‐richness in plant communities: The importance of the regeneration niche. Biological Reviews of the Cambridge Philosophical Society, 52, 107–145.

[ece310835-bib-0024] Hangelbroek, H. H. , & Santamaria, L. (2004). Regulation of propagule size in the aquatic pseudo‐annual *Potamogeton pectinatus*: Are genetic and maternal non‐genetic effects additive? Evolutionary Ecology Research, 6(1), 147–161.

[ece310835-bib-0025] Hijmans, R. J. , Cameron, S. E. , Parra, J. L. , Jones, P. G. , & Jarvis, A. (2005). Very high resolution interpolated climate surfaces for global land areas. International Journal of Climatology, 25(15), 1965–1978. 10.1002/joc.1276

[ece310835-bib-0026] Hodel, R. G. J. , Knowles, L. L. , McDaniel, S. F. , Payton, A. C. , Dunaway, J. F. , Soltis, P. S. , & Soltis, D. E. (2018). Terrestrial species adapted to sea dispersal: Differences in propagule dispersal of two Caribbean mangroves. Molecular Ecology, 27(22), 4612–4626. 10.1111/mec.14894 30308703

[ece310835-bib-0027] Jiang, X. (2014). Xylem hydraulic structure and function in mangroves. Ph.D. Dissertation. Guangxi University, Guangxi. 10.27034/d.cnki.ggxiu.2021.001491

[ece310835-bib-0028] Joshi, A. C. (1933). A suggested explanation of the prevalence of vivipary on the sea‐shore. Journal of Ecology, 21(1), 209–212.

[ece310835-bib-0029] Kendall, B. E. , & Fox, G. A. (2003). Unstructured individual variation and demographic stochasticity. Conservation Biology, 17(4), 1170–1172. 10.1046/j.1523-1739.2003.02411.x 35701963

[ece310835-bib-0030] Krauss, K. W. , Lovelock, C. E. , Mckee, K. L. , Lopez‐Hoffman, L. , Ewe, S. M. L. , & Sousa, W. P. (2008). Environmental drivers in mangrove establishment and early development: A review. Aquatic Botany, 89(2), 105–127. 10.1016/j.aquabot.2007.12.014

[ece310835-bib-0031] Kumordzi, B. B. , Aubin, I. , Cardou, F. , Shipley, B. , Violle, C. , Johnstone, J. , Anand, M. , Arsenault, A. , Bell, F. W. , Bergeron, Y. , Boulangeat, I. , Brousseau, M. , Grandpré, L. D. , Delagrange, S. , Fenton, N. J. , Gravel, D. , Macdonald, S. E. , Hamel, B. , Higelin, M. , … Munson, A. D. (2019). Geographic scale and disturbance influence intraspecific trait variability in leaves and roots of north American understorey plants. Functional Ecology, 33(9), 1771–1784. 10.1111/1365-2435.13402

[ece310835-bib-1002] Maguire, T. L. , Saenger, P. , Baverstock, P. , & Henry, R. (2000). Microsatellite analysis of genetic structure in the mangrove species *Avicennia marina* (Forsk.) Vierh. (Avicenniaceae). Molecular Ecology, 9(11), 1853–1862. 10.1046/j.1365-294x.2000.01089.x 11091321

[ece310835-bib-0032] Martin, A. R. , Rapidel, B. , Roupsard, O. , van den Meersche, K. , de Melo‐Virginio‐Filho, E. , Barrios, M. , & Isaac, M. E. (2017). Intraspecific trait variation across multiple scales: The leaf economics spectrum in coffee. Functional Ecology, 31(3), 604–612. 10.1111/1365-2435.12790

[ece310835-bib-0035] Moran, E. V. , Hartig, F. , & Bell, D. M. (2016). Intraspecific trait variation across scales: Implications for understanding global change responses. Global Change Biology, 22(1), 137–150. 10.1111/gcb.13000 26061811

[ece310835-bib-0036] Mounger, J. , Boquete, M. T. , Schmid, M. W. , Granado, R. , Robertson, M. H. , Voors, S. A. , Langanke, K. L. , Alvarez, M. , Wagemaker, C. A. M. , Schrey, A. W. , Fox, G. A. , Lewis, D. B. , Lira, C. F. , & Richards, C. L. (2021). Inheritance of DNA methylation differences in the mangrove *Rhizophora mangle* . Evolution and Development, 23(4), 351–374. 10.1111/ede.12388 34382741

[ece310835-bib-0037] Nettel, A. , & Dodd, R. S. (2007). Drifting propagules and receding swamps: Genetic footprints of mangrove recolonization and dispersal along tropical coasts. Evolution, 61(4), 958–971. 10.1111/j.1558-5646.2007.00070.x 17439624

[ece310835-bib-0038] Ouyang, S. , Xiang, W. H. , Wang, X. P. , Xiao, W. F. , Chen, L. , Li, S. G. , Sun, H. , Deng, X. W. , Forrester, D. I. , Zeng, L. X. , Lei, P. F. , Lei, X. D. , Gou, M. M. , & Peng, C. H. (2019). Effects of stand age, richness and density on productivity in subtropical forests in China. Journal of Ecology, 107(5), 2266–2277. 10.1111/1365-2745.13194

[ece310835-bib-0039] Paradis, E. , Claude, J. , & Strimmer, K. (2004). APE: Analyses of phylogenetics and evolution in R language. Bioinformatics, 20(2), 289–290. 10.1093/bioinformatics/btg412 14734327

[ece310835-bib-0040] Parida, A. K. , Das, A. B. , Sanada, Y. , & Mohanty, P. (2004). Effects of salinity on biochemical components of the mangrove, Aegiceras Corniculatum. Aquatic Ecology, 80(2), 77–87. 10.1016/j.aquabot.2004.07.005

[ece310835-bib-0041] Parida, A. K. , & Jha, B. (2010). Salt tolerance mechanisms in mangroves: A review. Trees‐Structure and Function, 24(2), 199–217. 10.1007/s00468-010-0417-x

[ece310835-bib-0042] Peterson, J. M. , & Bell, S. S. (2012). Tidal events and salt‐marsh structure influence black mangrove (*Avicennia germinans*) recruitment across an ecotone. Ecology, 93(7), 1648–1658. 10.1890/11-1430.1 22919911

[ece310835-bib-0043] Pinheiro, J. , Bates, D. , & R Core Team . (2022). nlme: Linear and Nonlinear Mixed Effects Models. R package version 3.1–158 [online]. https://CRAN.R‐project.org/package=nlme

[ece310835-bib-0045] R Core Team . (2022). R: A language and environment for statistical computing. R Foundation for Statistical Computing https://www.R‐project.org/

[ece310835-bib-0047] Richards, C. L. , Langanke, K. L. , Mounger, J. , Fox, G. A. , & Lewis, D. B. (2021). Trait response to nitrogen and salinity in *Rhizophora mangle* propagules and variation by maternal family and population of origin. Frontiers in Marine Science, 8, 756683. 10.3389/fmars.2021.756683

[ece310835-bib-0049] Rosseel, Y. (2012). Lavaan: An R package for structural equation modeling. Journal of Statistical Software, 48(2), 1–36. 10.18637/jss.v048.i02

[ece310835-bib-0050] Saatkamp, A. , Cochrane, A. , Commander, L. , Guja, L. K. , Jimenez‐Alfaro, B. , Larson, J. , Nicotra, A. , Poschlod, P. , Silveira, F. A. O. , Cross, A. T. , Dalziell, E. L. , Dickie, J. , Erickson, T. E. , Fidelis, A. , Fuchs, A. , Golos, P. J. , Hope, M. , Lewandrowski, W. , Merritt, D. J. , … Walck, J. L. (2019). A research agenda for seed‐trait functional ecology. New Phytologist, 221(4), 1764–1775. 10.1111/nph.15502 30269352

[ece310835-bib-0051] Saenger, P. , & West, P. W. (2018). Phenotypic variation of the mangrove species *Avicennia marina* (Forssk.) Vierh. From seven provenances around Australia. Aquatic Ecology, 149, 28–32. 10.1016/j.aquabot.2018.05.004

[ece310835-bib-0052] Sheue, C. R. , Liu, H. Y. , & Yong, J. W. H. (2003). *Kandelia obovata* (Rhizophoraceae), a new mangrove species from eastern Asia. Taxon, 52(2), 287–294. 10.2307/3647398

[ece310835-bib-0053] Sousa, W. P. , Kennedy, P. G. , Mitchell, B. J. , & Ordóñez, L. B. M. (2007). Supply‐side ecology in mangroves: Do propagule dispersal and seedling establishment explain forest structure? Ecological Monographs, 77(1), 53–76. 10.1890/05-1935

[ece310835-bib-0054] Tomlinson, P. (2016). The botany of mangroves (2nd ed.). Cambridge University Press. 10.1017/CBO9781139946575

[ece310835-bib-0055] Van der Stocken, T. , De Ryck, D. J. R. , Vanschoenwinkel, B. , Deboelpaep, E. , Bouma, T. J. , Dahdouh‐Guebas, F. , & Koedam, N. (2015). Impact of landscape structure on propagule dispersal in mangrove forests. Marine Ecology Progress Series, 524, 95–106. 10.3354/meps11206

[ece310835-bib-0056] Van der Stocken, T. , Wee, A. K. S. , De Ryck, D. J. R. , Vanschoenwinkel, B. , Friess, D. A. , Dahdouh‐Guebas, F. , Simard, M. , Koedam, N. , & Webb, E. L. (2019). A general framework for propagule dispersal in mangroves. Biological Reviews, 94(4), 1547–1575. 10.1111/brv.12514 31058451

[ece310835-bib-0057] Vasey, G. L. , Weisberg, P. J. , & Urza, A. K. (2022). Intraspecific trait variation in a dryland tree species corresponds to regional climate gradients. Journal of Biogeography, 49(12), 2309–2320. 10.1111/jbi.14515

[ece310835-bib-0058] Violle, C. , Enquist, B. J. , McGill, B. J. , Jiang, L. , Albert, C. H. , Hulshof, C. , Jung, V. , & Messier, J. (2012). The return of the variance: Intraspecific variability in community ecology. Trends in Ecology and Evolution, 27(4), 244–252. 10.1016/j.tree.2011.11.014 22244797

[ece310835-bib-0059] Wang, W. Q. , You, S. Y. , Wang, Y. B. , Huang, L. , & Wang, M. (2011). Influence of frost on nutrient resorption during leaf senescence in a mangrove at its latitudinal limit of distribution. Plant and Soil, 342(1–2), 105–115. 10.1007/s11104-010-0672-z

[ece310835-bib-0060] Wu, L. W. , Zhang, Y. , Guo, X. , Ning, D. L. , Zhou, X. S. , Feng, J. J. , Yuan, M. M. , Liu, S. , Guo, J. J. , Gao, Z. P. , Ma, J. , Kuang, J. L. , Jian, S. Y. , Han, S. , Yang, Z. F. , Ouyang, Y. , Fu, Y. , Xiao, N. J. , Liu, X. D. , … Zhou, J. Z. (2022). Reduction of microbial diversity in grassland soil is driven by long‐term climate warming. Nature Microbiology, 7, 1054–1062. 10.1038/s41564-022-01147-3 35697795

[ece310835-bib-0061] Wu, Y. T. , Ricklefs, R. E. , Huang, Z. J. , Zan, Q. J. , & Yu, S. X. (2018). Winter temperature structures mangrove species distributions and assemblage composition in China. Global Ecology and Biogeography, 27(12), 1492–1506. 10.1111/geb.12826

[ece310835-bib-0062] Yang, S. , Liu, X. , Deng, R. J. , Chen, Q. X. , Wang, J. W. , & Lu, X. (2020). Geographic variations of hypocotyl and seedling growth traits for *Kandelia obovata* with different provenances. Chinese Journal of Ecology, 39(6), 1769–1777. 10.13292/j.1000-4890.202006.003

[ece310835-bib-0063] Zhang, Y. , Xin, K. , Sheng, N. , Xie, Z. L. , & Liao, B. W. (2021). The regenerative capacity of eight mangrove species based on propagule traits in Dongzhai Harbor, Hainan Province, China. Global Ecology and Conservation, 30, e01788. 10.1016/j.gecco.2021.e01788

[ece310835-bib-1005] Zhou, X. X. , Cai, L. L. , Fu, M. P. , Hong, L. W. , Shen, Y. J. , & Li, Q. Q. (2016). Progress in the studies of vivipary in mangrove plants. Chinese Journal of Plant Ecology, 40(12), 1328–1343. 10.17521/cjpe.2016.0087

[ece310835-bib-0064] Zhu, H. , Lin, H. J. , Yang, L. , Li, H. P. , Yue, C. L. , & Jiang, B. (2021). Geographical distribution pattern and environmental explanation of *Kandelia obovata* Sheue, H. Y. Liu & J. Yong populations along the southeast coast of China. Plant Science Journal, 39(5), 476–487. 10.11913/PSJ.2095-0837.2021.50476

